# The tripeptide feG inhibits leukocyte adhesion

**DOI:** 10.1186/1476-9255-5-6

**Published:** 2008-05-20

**Authors:** Ronald D Mathison, Emily Christie, Joseph S Davison

**Affiliations:** 1 University of Calgary, Faculty of Medicine, Department of Physiology and Biophysics, 3330 Hospital Drive NW, Calgary Alberta T2N 4N1, Canada

## Abstract

**Background:**

The tripeptide feG (D-Phe-D-Glu-Gly) is a potent anti-inflammatory peptide that reduces the severity of type I immediate hypersensitivity reactions, and inhibits neutrophil chemotaxis and adhesion to tissues. feG also reduces the expression of β1-integrin on circulating neutrophils, but the counter ligands involved in the anti-adhesive actions of the peptide are not known. In this study the effects of feG on the adhesion of rat peritoneal leukocytes and extravasated neutrophils to several different integrin selective substrates were evaluated.

**Results:**

The adhesion of peritoneal leukocytes and extravasated neutrophils from rats to adhesive proteins coated to 96-well plates was dependent upon magnesium (Mg^2+^) ion, suggestive of integrin-mediated adhesion. feG inhibited leukocyte adhesion, but only if the cells were stimulated with PAF (10^-9^M), indicating that feG's actions *in vitro *require cell activation. In the dose range of 10^-10^M to 10^-12^M feG inhibited the adhesion of peritoneal leukocytes to fibrinogen and fibronectin, but not IgG, vitronectin or ICAM-1. feG inhibited the binding of extravasated neutrophils to heparin, IgG, fibronectin and CD16 antibody. Antigen-challenge of sensitized rats reduced the adhesion of peritoneal leukocytes to most substrates and abolished the inhibitory effects of feG. However, pretreating the animals with intraperitoneal feG (100 μg/kg) 18 h before collecting the cells from the antigen-challenged animal restored the inhibition of adhesion by *in vitro *feG of peritoneal leukocytes and extravasated neutrophils to fibronectin.

**Conclusion:**

The modulation of leukocyte adhesion by feG appears to involve actions on αMβ2 integrin, with a possible interaction with the low affinity FcγRIII receptor (CD16). The modulation of cell adhesion by feG is dual in nature. When administered *in vivo*, feG prevents inflammation-induced reductions in cell adhesion, as well as restoring its inhibitory effect *in vitro*. The mechanism by which *in vivo *treatment with feG exerts these effects remains to be elucidated.

## Background

The tripeptide FEG (Phe-Glu-Gly) was isolated from rat submandibular glands as a component of a heptapeptide (SGPT) located at the C-terminal end transcript of the variable coding sequence a1 gene (*Vcsa1*; also known as a submandibular rat 1 gene; (SMR1)) [1,2]. The D-isomer of FEG (feG), is active in all species tested to date including mice [3], sheep [4], cats [5], dogs and isolated human neutrophils [6]. The peptide has potent anti-inflammatory action that effectively reduces allergic or type I immediate hypersensitivity reactions, as revealed by attenuated vascular leakage, intestinal motility disturbances, systemic hypotension, bronchoconstriction and hyper-reactivity, and pulmonary inflammation [4,7-10]. Additionally, these peptides modulate neutrophil function by inhibiting their adhesion, chemotaxis, and production of intracellular superoxide [6,11-13].

By interfering with leukocyte adhesion and chemotaxis, feG arrests the movement of cells into the extravascular space and prevents their migration to the site of inflammation [7,14], thereby reducing the severity of the inflammation. Some of the anti-adhesive actions of feG stem from the peptide modifying the expression of integrins and the binding properties of the integrin-associated IgG receptor – CD16 (FcγRIII) [6,10,12]. The integrins, heterodimeric cell surface receptors involved in diverse biological responses from embryonic development, thrombosis, and immune and inflammatory responses, are essential players in the adhesion, extravasation and migration of leukocytes [15,16].

The objective of this study was to further characterize the specificity of feG's inhibitory action on leukocyte adhesion by examining adhesion to substrates that show selectivity for the integrins expressed on neutrophils. These include: highly expressed β2-integrins, αLβ2 (CD11a/CD18) and αMβ2 (CD11b/CD18), and others that are poorly expressed, such as αXβ2 (CD11c/CD18), α2β1 (CD49b/CD29), α4β1 (CD49d/CD29), α5β1(CD49e/CD29), and αVβ3 (CD51/CD61) [17-19]. α4β1 is of interest because its expression is up-regulated on activated neutrophils [12,17-19].

## Methods

### Animal groups and sensitization

The protocols for all animal experiments were approved by the University of Calgary Health Sciences Animal Care Committee, which conforms to the guidelines of the Canadian Council on Animal Care. Male Sprague-Dawley rats (Charles River Canada, Saint-Constant, QC), of an initial weight or 160–175 g, were housed under controlled lighting conditions (lights on from 7:00 H to 19:00 H), and provided with food and water *ad libitum*. Previous studies have established that feG does not affect leukocyte function in normal animals or cells, but its effects are revealed upon imposition of an inflammatory stimulus [6,7,10,20]. Thus, several groups of animals were used that included: 1) normal, unsensitized rats; 2) unsensitized rats treated with 100 μg/kg feG 18 h before harvesting the cells; 3) ovalbumin (OA)-sensitized rats challenged with antigen 18 h before harvesting cells; and 4) ovalbumin-sensitized rats challenged with antigen and treated with 100 μg/kg feG 18 h before harvesting cells. feG has a half-life of approximately 12 h [21], and in several studies pre-treatment with feG 18 h before leukocyte isolation has demonstrated attenuated inflammatory responses to endotoxin and allergen [12,14].

Rats were sensitized with an intraperitoneal injection of 1 mg OA and 50 ng pertussis toxin (Sigma Chemical, St. Louis, Mo.) as adjuvant: a sensitization method generating elevated IgE titres [22,23]. The animals were used 28 to 35 days post-sensitization. Rats received oral antigen by gastric lavage with 100 mg/kg of OA in 0.9% saline, whereas unchallenged sensitized animals received a neutral antigen, bovine serum albumin (BSA).

### Leukocyte preparation

Leukocytes were obtained from three sources: blood, the peritoneal cavity or a carrageenan-soaked, implanted sponge. Underhalothane anaesthesia 9–10 mL of blood was collected by cardiac puncture into a 12 mL syringe, containing 1 ml of 3.8% Na citrate, an anticoagulant. The blood (10–12 mL) was diluted with polymorphonuclear leukocyte (PMN) buffer without calcium to 50 mL in a polypropylene centrifuge tube, and centrifuged at 400 g for 15 min at 4°C. The PMN buffer was of the following composition: 138 mM NaCl, 2.7 mM KCl, 3.2 mM Na_2_HPO_4_.12H_2_O, 5.5 mM glucose. The white blood cells were removed from the surface of the pellet with a plastic Pasteur pipette, and contaminating red blood cells were lysed with 4 volumes of 0.15 M NH_4_Cl for 10 min at room temperature. The volume of the polypropylene centrifuge tube was completed to 50 mL with PMN buffer without calcium, and after a second spin at 400 g for 10 min at 4°C, the supernatant was discarded. The pellet was washed with calcium free PMN buffer and centrifuged again at 400 g for 10 min at 20°C. The supernatant was discarded and the cells resuspended in 1 mL of PMN buffer containing calcium (1.2 mM CaCl_2_), magnesium (1.5 mM MgCl_2_).

Peritoneal cells were obtained by injecting 10 ml of 0.9% saline into the peritoneum, and after massaging, a laparotomy was performed and the saline aspirated with a plastic Pasteur pipette. The cells were washed twice in calcium free PMN buffer as described for the blood cells before resuspending them in Ca^2+-^PMN buffer.

Extravasated neutrophils were collected by placing, under halothane anaesthesia, a small sponge soaked in 0.5% carrageenan subcutaneously into the intrascapular region [24]. To implant the sponge a 2–3 cm incision was made dorsally, between the shoulder blades, and connective tissue was cleared from the exposed area. The skin was then closed with sutures of 3-0 Dexon thread. Eighteen hours later the sponge was removed and the fluid was squeezed from it into 5 mLs of PMN buffer. Following centrifugation at 400 g for 10 min, the exudate was decanted and the remaining cells were suspended in Ca^2+^-PMN buffer. Total leukocyte counts were determined with a Hylite hemocytometer (Hauser Scientific, Boulder, CO) using Trypan Blue exclusion as a marker of cell viability.

### Adhesion assay

Leukocyte adhesion was performed using modifications of a crystal violet assay [25-27]. The wells of 96-cell polystyrene Nunclon plates (Nalge Nunc International, Naperville, IL) were coated with various substrate molecules, using literature cited amounts – fibrinogen (500 ng/ml; [28]), fibronectin (2.5 μg/ml; [29]), rat serum IgG (10 μg/ml; [30]), or vitronectin (350 ng/ml; [31]). The plates were allowed to dry at room temperature overnight, then washed twice with 0.9% saline, dried again and then stored at 4°C until use within 2–3 weeks. Leukocytes(2.5 × 10 ^5 ^in 200 μl of PMN buffer) were distributed into the protein-coated wells and different final concentrations (10^-10^M to 10^-12^M) of the peptide feG were added (10 μL) to separate wells. The cells were allowed to adhere for 45 min at 37°C. The wells were then washed 3 times in 200 μl of PMN buffer, fixed with 10% formalin for 10 min, before adding crystal-violet (crystal-violet 7·5 g/l, NaCl 2·5 g/l, formaldehyde 1·57%, methanol 50%) for an additional 5 min. The cells were washed 3 times with distilled water, solubilized with 10% sodium dodecyl sulphate (SDS), and the plates were read at 540 nm (Multiskan Ascent, Thermo Scientific, Waltham, MA). After subtraction of non-specific colorimetric readings to obtain absolute binding, the percent inhibition of leukocyte adhesion by feG was calculated relative to the wells containing only PAF (10^-9^M).

The adhesion of neutrophils to various antibodies to various cell adhesion molecules was evaluated using anti-rat CD11b (clone OX-42; isotype – IgG2a; BD-Pharmingen, San Diego, CA); mouse anti-rat CD18 (clone WT.3; isotype – IgG1; AbD Serotec, Cedarlane Laboratories Ltd; Hornby ON); mouse anti-Rat CD32 (Clone: D34-485; isotype – IgG1; RDI Research Diagnostics Inc., Concord MA); hamster anti-rat CD62L (Clone: HRL1; isotype – IgG2a; BD-Pharmingen) and anti-human CD16 (clone LNK16; isotype – IgG1; Advanced ImmunoChemical Inc, Long Beach, CA). 0.1 μg of antibody was added to each well of a 96-well plate [32], and the adhesion study performed as described above.

### Peptides and Chemicals

feG was synthesized by American Peptide Co., Sunnyvale, CA. Platelet activating factor PAF(C_16_) (1-Hexadecyl-2-acetyl-*sn*-glycero-3-phosphocholine), obtained from Sigma-Aldrich, St. Louis was dissolved in 100% ethanol at a concentration of 10^-2^M and stored at -20°C in 5 μl aliquots, and diluted 10^7 ^fold for use at a final concentration of 10^-9^M. Rat tail collagen, IgG from rat sera, vitronectin from human plasma were purchased from Sigma-Aldrich. Fibrinogen (plasminogen-depleted from human plasma) was obtained from Calbiochem, San Diego, CA. Fibronectin (human) BD Biosciences, San Jose, CA Recombinant human soluble ICAM-1 from Bender MedSystems Inc. Burlingame, CA. Heparin was from Organon Canada Ltd. Toronto, ON.

### Statistical analysis

The results are presented as the mean ± SEM. The statistical functions used are associated with Excel (Microsoft Office XP, Redmond, WA). Comparisons between treatment groups were made with one-way analysis of variance (ANOVA), and if warranted differences between two groups were evaluated using the unpaired Student's t-test. Statistical values reaching probabilities of p < 0.05 were considered significant.

## Results

### General characteristics of leukocyte adhesion

Adhesion of circulating and peritoneal leukocytes, as well as extravasated neutrophils, to fibrinogen and fibronectin increased significantly when magnesium ion (Mg^2+^) was present in the buffer. The adhesion of blood leukocytes, predominately monocytes/lymphocytes, was at least 50% less than that of peritoneal cells (macrophages and neutrophils) and extravasated neutrophils. Due to this low adhesion of blood leukocytes the effects of feG on adhesion were evaluated using peritoneal leukocytes and extravasated neutrophils.

**Figure 1:**
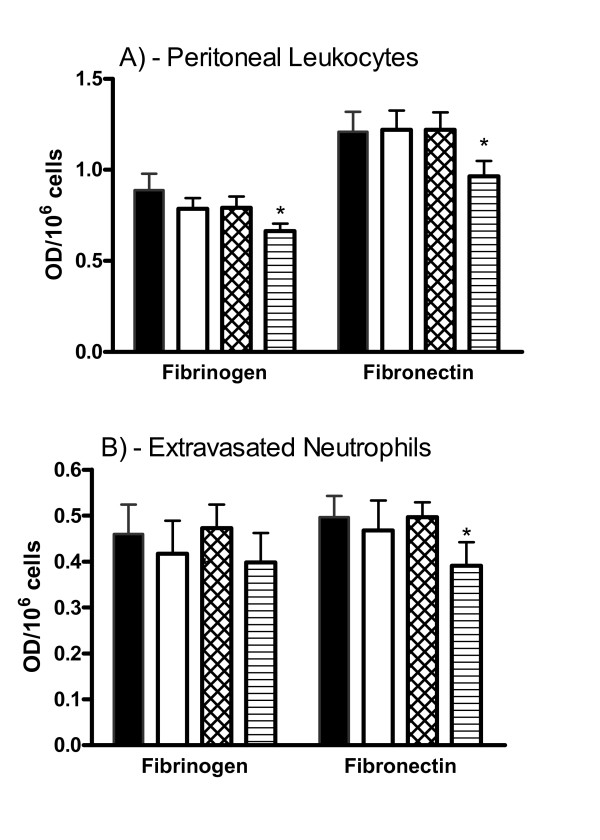
**PAF is required for inhibition of adhesion by feG**. The inclusion of PAF (10^-9^M) was required to observe an inhibitory effect of feG (10^-11^M) on the adhesion of peritoneal leukocytes from unsensitized animals to fibrinogen and fibronectin. With extravasated neutrophils feG inhibition of adhesion only occurred in the presence of PAF for fibronectin. Control (black column), feG (open column; 10^-11^M), PAF (crossed column), and PAF + feG (lined column). Mean ± SEM; p < 0.05; * = less than corresponding controls; n ≥ 4 for each group of cells and substrate.

In a previous study stimulation with PAF (10^-9^M) was required to observe an inhibitory effect of feG both on rat leukocyte adhesion to atrial tissue [20], and inhibition of CD16 antibody binding to human neutrophils [6]. This requirement for PAF also was observed for feG (10^-11^M) inhibition of adhesion of peritoneal leukocytes from unsensitized animals to fibrinogen and fibronectin (Figure 1A). Similar results were seen with extravasated neutrophils adhesion to fibronectin (Figure 1B), although feG did not inhibit the adhesion of these cells to fibrinogen (Figure 1B) or IgG (not shown). Extravasated neutrophils did not bind to collagen. For all subsequent experiments PAF was included in the adhesion assay.

### Effects of sensitization and an allergic reaction

The presence of an allergic response in the sensitized rats was established by monitoring differential cell counts in blood [12]. Antigen challenge of sensitized animals caused a circulating neutrophilia (48.7 ± 4.4% of circulating white blood cells) that was ~2.5 times greater than that of unsensitized animals (19.2 ± 2.9%). Treatment with feG did not alter white blood cell counts in unsensitized animals, but effectively prevented the neutrophilia occurring in sensitized animals (28.9 ± 3.4%).

Changes, reported below, in cell adhesion with sensitized animals were not due to differential cell numbers in the peritoneal lavage fluid or in the carrageenan-soaked sponge, since peritoneal lavage fluid contained 11 to 12% neutrophils and 35 to 43% macrophages and was the same in the 4 animal groups studied. The carrageenan-soaked sponge cells were >99% neutrophils in all animal groups.

**Figure 2:**
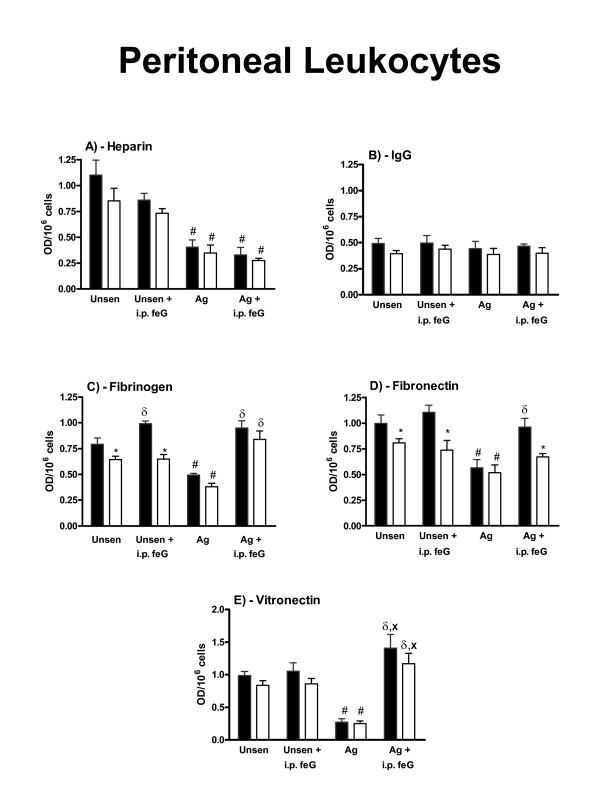
**Effects of feG on the adhesion of peritoneal leukocytes to heparin, IgG, fibrinogen, fibronectin and vitronectin**. Three different types of results are summarized: 1) the effects of antigen challenge (Ag); 2) the effects of *in vivo *treatment with feG (i.p. feG); and 3) the effects of *ex vivo *feG (open column). The adhesion, expressed as optical density (O.D.) for 1 million cells, is shown in the presence of PAF (10^-9^M) alone (black column), and in the presence of PAF and *ex vivo *feG (open column). The adhesion in the presence of *ex vivo *feG is the average adhesion for 10^-10^M to 10^-12^M feG added to the cells in the 96-well plate bars. Results are shown for four groups of animals: unsensitized (Unsens); unsensitized treated intraperitoneally with feG 18 h before harvesting the cells (Unsens + i.p. feG); antigen-challenged sensitized (Ag); and antigen-challenged sensitized treated intraperitoneally with feG 18 h before harvesting the cells (Ag + i.p. feG). Mean ± SEM; p < 0.05. # less than corresponding unsensitized group; χ greater than unsensitized group; * less than corresponding control (PAF alone); δ greater than no i.p. feG; X greater than corresponding unsensitized group. n ≥ 4 for each group of cells.

**Figure 3:**
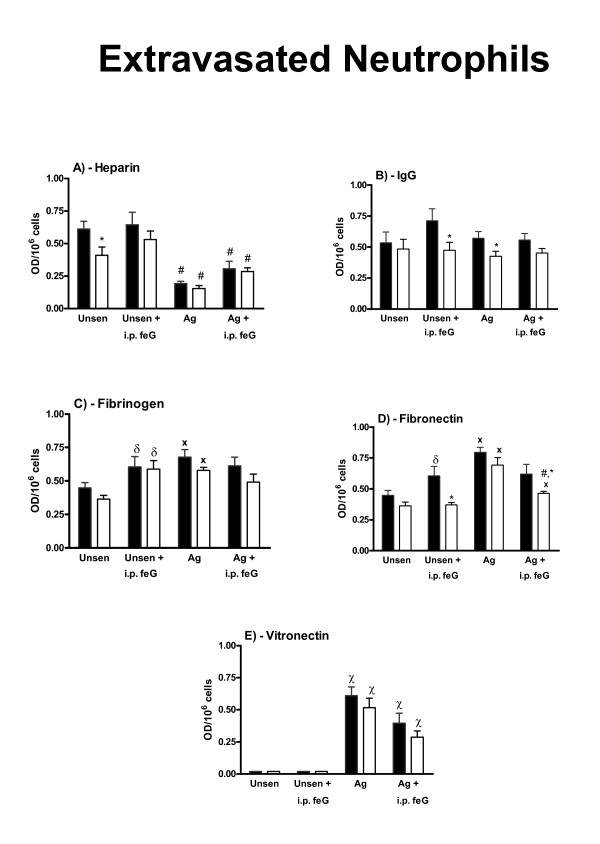
**Effects of feG on the adhesion of carrageenan neutrophils to heparin, IgG, fibrinogen, fibronectin and vitronectin**. Three different types of results are summarized: 1) the effects of antigen challenge (Ag); 2) the effects of *in vivo *treatment with feG (i.p. feG); and 3) the effects of *ex vivo *feG (open column). The adhesion, expressed as optical density (O.D.) for 1 million cells, is shown in the presence of PAF (10^-9^M) alone (black column), and in the presence of PAF and *ex vivo *feG (open column). The adhesion in the presence of *ex vivo *feG is the average adhesion for 10^-10^M to 10^-12^M feG added to the cells in the 96-well plate bars. Results are shown for four groups of animals: unsensitized (Unsens); unsensitized treated intraperitoneally with feG 18 h before harvesting the cells (Unsens + i.p. feG); antigen-challenged sensitized (Ag); and antigen-challenged sensitized treated intraperitoneally with feG 18 h before harvesting the cells (Ag + i.p. feG). Mean ± SEM; p < 0.05. # less than corresponding unsensitized group; χ greater than unsensitized group; * less than corresponding control (PAF alone); δ greater than no i.p. feG; X greater than corresponding unsensitized group. n ≥ 4 for each group of cells.

When cells were collected from sensitized animals that were not challenged with antigen, the adhesion of peritoneal leukocytes to any of the substrates was not significantly different from that seen with unsensitized animals (not shown). However, when peritoneal leukocytes were collected from antigen-challenged animals, lower adhesion to heparin, fibrinogen, fibronectin and vitronectin (Figures 2A, C, D and 2E) but not to IgG (Figure 2B) was observed. Treatment with feG (100 μg/kg) at the time of antigen challenge reversed this antigen-induced reduction in adhesion to these substrates except for heparin. The adhesion of extravasated neutrophils to IgG and fibrinogen was not affected by antigen challenge (Figures 3B and 2C), although adhesion to heparin of the extravasated cells was reduced (Figure 3A) and adhesion to fibronectin was increased. With unsensitized animals the extravasated neutrophils did not adhere to vitronectin (Figure 3E), although antigen challenge of the sensitized animals resulted in significant adhesion to vitronectin.

### Effects of *ex vivo *feG on leukocyte adhesion

**Figure 4:**
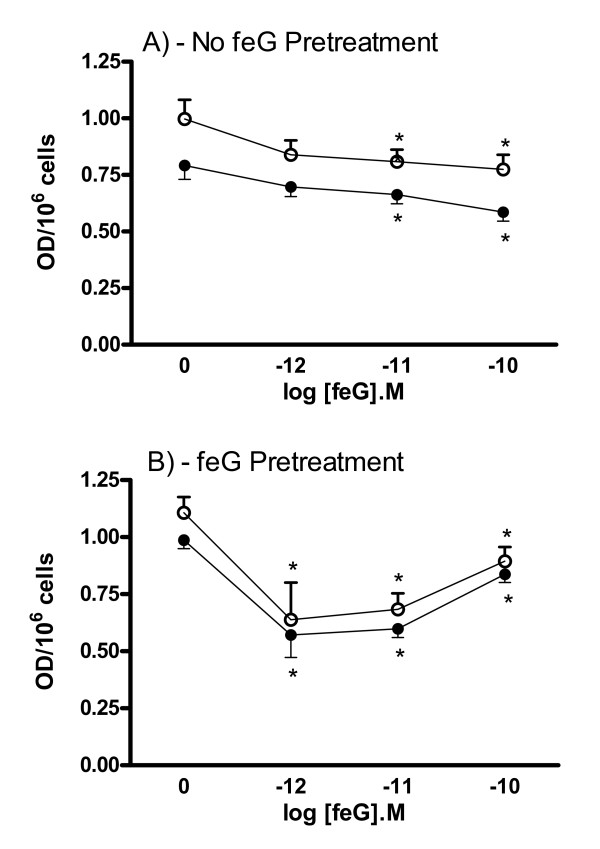
**Dose-dependent inhibition of peritoneal leukocyte adhesion to fibrinogen and fibronectin**. The inhibition of peritoneal leukocyte adhesion (OD/10^6 ^cells) to fibrinogen (black circles) and fibronectin (open circles) by 10^-10^M, 10^-11^M and 10^-12^M feG is shown for cells obtained from unsensitized rats that were either untreated (A) or pretreated with 100 μg/kg feG intraperitoneally 18 h before collecting the cells (B). Mean ± SEM; p < 0.05. * less than no feG *in vitro *(0). N ≥ 4.

Figure 4 shows dose response relationships (10^-10^M to 10^-12^M; [6,12]) for the inhibitory effect of feG on PAF-stimulated peritoneal leukocytes from unsensitized rats that were not pretreated with feG (Figure 4A), and those that received intraperitoneal feG (100 μg/kg) 18 hr before isolating the cells (Figure 4B). With cells from animals that were not pretreated with feG (Figure 4A), the *ex vivo *addition of feG to the assay wells at 10^-10^M and 10^-11^M inhibited leukocyte adhesion to fibrinogen by 24.2 ± 3.7% and 14.3 ± 4.4%, respectively, and to fibronectin by 17.3 ± 8.4% and 16.0 ± 5.3%, respectively. Peritoneal leukocytes from unsensitized animals avidly bound to ICAM-1 (OD of 2.36 ± 0.08/10^6 ^cells), although feG did not affect the adhesion to this substrate (not shown).

In contrast, with feG pretreatment (Figure 4B), the inhibition of adhesion of peritoneal leukocytes to fibronectin and fibrinogen increased significantly to an average of 32.0 ± 7.5% and 31.7 ± 6.1%, respectively, for the three concentrations of feG. A sensitization of the leukocytes to the inhibitory effect of *ex vivo *feG occurred as the significant inhibition of adhesion seen with 10^-12^M peptide was absent if the animals were not pretreated with feG.

The inhibitory effects of *ex vivo *feG on peritoneal leukocyte adhesion to fibrinogen and fibronectin were abolished when sensitized animals were challenged with antigen (Figure 2). However, the *in vivo *pretreatment with feG re-established the inhibitory effect of feG on adhesion to fibronectin, but not fibrinogen (Figure 2C and 2D).

With extravasated neutrophils from unsensitized animals *ex vivo *feG only inhibited adhesion to heparin (Figure 3A), and with antigen-challenged animals inhibition of adhesion of these cells to IgG occurred (Figure 3B). Pretreatment with feG enabled an inhibitory effect of *ex vivo *feG on extravasated neutrophil adhesion to IgG in unsensitized rats (Figure 3B) and fibronectin with sensitized rats (Figure 3D).

### Effects of feG on leukocyte adhesion to antibodies

**Figure 5:**
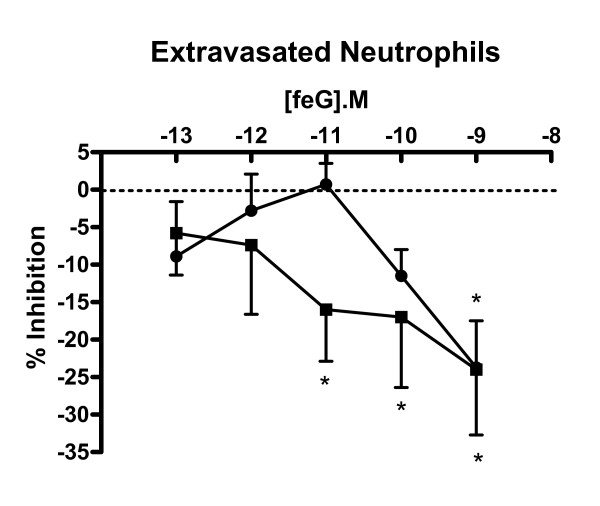
**Dose-dependent inhibition of neutrophil adhesion to antibodies**. Inhibition by feG of the adhesion of extravasated neutrophils to CD11b (black circle) and CD16b (black square). Mean ± SEM; p < 0.05; * = significant inhibition. n ≥ 7 for each antibody and the different doses of feG.

Since feG inhibits the binding of CD11b and CD16b antibody to human neutrophils [6], the effects of feG on the adhesion of extravasated neutrophils to integrin antibodies and CD16b were evaluated. feG did not modify neutrophil adhesion to CD18b (β2 integrin), CD62L (L-selectin) or CD32 (FcγRII; intermediate affinity IgG receptor) (not shown). feG modestly, but dose-dependently, inhibited the adhesion of neutrophils to human CD16 (FcγRIII; intermediate affinity IgG receptor) antibody, and at the highest dose tested (10^-9^M) inhibited neutrophil adhesion to CD11b antibody by 24 % (Figure 5). The inhibition of adherence to CD11b and CD16 antibodies by feG is not due to non-specific binding effects as anti-CD62L was the same isotype as anti-CD11b (IgG2a), and anti-CD18 and anti-CD32 were the same isotype (IgG1) as CD16.

## Discussion

In keeping with other studies using human neutrophils [33,34], we found that the adhesion of rat leukocytes required the presence of Mg^2+ ^ion in the incubating buffer, indicating that leukocyte adhesion is mediated by an integrin possessing a metal ion-dependent adhesion site (MIDAS). This Mg^2+^/Mn^2+ ^binding site is located in the I domain of seven integrin α-subunits (α1, α2, αL, αM, αX, αD, αV and αE) [35]. The requirement of cell stimulation with PAF (Figure 1) for feG to inhibit adhesion reflects previous results showing that leukocytes activation was essential for feG to inhibit cell adhesion to atrial tissue [20], binding of CD16 antibody to neutrophils [6] and superoxide production [12]. Although the molecular basis for this activation requirement for an effect of feG is not known functional activation by pro-inflammatory mediators with resulting changes in integrin affinity is a common feature of integrin-mediated actions [36,37].

The tripeptide feG was found to inhibit leukocyte adhesion to several integrin-selective substrates, although the identity of the specific integrin was not conclusively identified. The inhibition of adhesion of peritoneal leukocytes to fibrinogen is indicative of modification of αMβ2 integrin-mediated adhesion. Leukocytes can adhere to fibrinogen by using αMβ2, αXβ2 and αVβ3 integrins [15,38]. However, since feG did not modify adhesion to vitronectin, an αVβ3 integrin selective substrate [39], nor adhesion to collagen, which interacts preferentially with αXβ2 (CD11c), α1β1, α2β1, α10β1 and α11β1[40,41] and αXβ2 is generally, with some exceptions [42,43], poorly expressed on neutrophils, feG's probably alters αM-mediated adhesion. An apparent anomalous observation is that feG did not modify adhesion to ICAM-1 which also interacts with αMβ2 [15]. However, the binding sites on αMβ2 for ICAM-1 and fibrinogen are distinct [44,45], and other αMβ2 integrin inhibitors block adhesion to fibrinogen but not to ICAM-1 [46].

An apparent exception to the selectivity of feG interfering with αMβ2-mediated adhesion is the inhibition by this peptide of leukocyte and neutrophil adhesion to fibronectin, which is generally considered to adhere to four integrins (α3β1, α4β1, α5β1, αVβ3) using the RGD (arginine-glycine-aspartic acid) motif [38]. However, several studies have shown that αMβ2 integrin binds to fibronectin via coordinate interactions with β1 integrins [47,48]. This interaction may involve initial engagement of β1 integrins on neutrophils with a resulting cross-talk signal leading to activation of αMβ2-mediated adhesion [48]. Thus, feG probably interacts or modifies a restricted subset of binding sites on the versatile and promiscuous αMβ2 integrin [38]. Cross-talk between α4β1 and αVβ3 also occurs [49], and may account for the trend towards inhibitory actions of *ex vivo *feG on extravasated neutrophils binding to vitronectin (Figure 4E).

A previously proposed role for FcγRIII in the actions of feG [6,50] is supported by the observation that the peptide inhibited the adhesion of extravasated neutrophils to a CD16 antibody (Figure 5), and IgG (Figure 3B). αMβ2 is known to cooperate with FcγRIII for the internalization of IgG-coated particles [51] and the generation of a respiratory burst [52]. In these cells, a physical proximity and association exists between CD16 and αMβ2 integrin [53,54]. The absence of an effect of feG on peritoneal leukocyte adhesion to IgG may reflect transient binding observed for human circulating neutrophils [30].

It is not known whether feG, which has its origins in the salivary glands of rats [2,55], acts as hormonal regulator of integrin-mediated adhesive interactions, or reflects a binding motif on an integrin or an integrin ligand. Most adhesive interactions between ligands and their substrates involve "a substrate recognition sequences" [38]. A FEG-like motif does not exist in IgG, fibrinogen or fibronectin, thus feG is probably not acting as "substrate recognition motif" to prevent integrin-substrate interactions. A FEG-like motif (FEA at F302-A304) is found on the α7 tail of αM integrin, and this sequence deserves attention as contributing to αM integrin-mediated adhesion. Exogenous FEG may be acting as a mimic of this α7 tail motif. Although a FEG sequence is found on laminins, tenascin C and versican, it is not known if this motif in these proteins serves as a recognition site for adhesive events, which are generally considered to be mediated by the RGD motif for laminin interactions with several integrin heterodimers (α1β1, α2β1, α3β1, α6β1, α7β1 and α6β4) [56], as is the case for tenascin interactions with α5β1[57]. Moreover, αMβ2 integrins do not play a major role in the adhesion of leukocytes to these extracellular matrix molecules [31].

The low adhesion of mixed blood leukocytes from rats to the fibrinogen and fibronectin precluded their study, and probably reflects the high proportion of lymphocytes/monocytes in rat blood, which only exhibit significant adhesion when stimulated with cytokines [58], or to more complex substrates such as heart tissue or cultured epithelial and endothelial cells [20,58,59]. The reduced adhesion of peritoneal leukocytes from antigen-challenged rats relative to unsensitized rats (Figure 2) may reflect a state of unresponsiveness or phenotypic modification of the cells resulting from the activation of the immediate hypersensitivity reaction. A similar loss of response by neutrophils is seen in other pathologies, such as portal hypertension, sepsis and severe injury [60-62]. Pretreatment with feG (18 h before cell collection) prevents the development of reduced adhesion in antigen-challenged animals, as is also seen with the increased production of intracellular superoxide by circulating neutrophils of antigen-challenged sensitized rats [12]. The reduced adhesion of peritoneal leukocytes was not due to lower expression of CD11b, since the surface expression of this integrin was increased by antigen challenge, and feG pre-treatment prevented this increase (unpublished observations). In what appears to be a paradox, antigen challenge had the opposite effect on extravasated neutrophils, enhancing their adhesion to fibronectin and vitronectin (Figure 3), indicating that feG may have differential actions depending upon the source of the cells, and possibly cross-talk interactions between integrins discussed above. The basis of these differences and interactions should become clear once the mechanism of action of feG is elucidated.

## Conclusion

The tripeptide feG, an anti-inflammatory peptide, may inhibit leukocyte adhesion by interfering with αMβ2 integrin-mediated adhesion. Several facets to feG's actions exist: an acute *ex vivo *inhibitory effect; and when the peptide is administered *in vivo*, a prevention of loss of peritoneal leukocyte binding in antigen-challenged animals with restoration of *ex vivo *inhibition.

## List of abbreviations

feG: D-phenylalanine-D-glutamate-Glycine; SGPT: submandibular gland peptide-T (Thr-Asp-Ile-Phe-Glu-Gly-Gly)

## Competing interests

RM and JSD have shares in a privately held company that is developing analogues of the tripeptide feG for therapeutic use.

## Authors' contributions

All authors participated in study design and read and approved the final manuscript. JSD aided in protocol development and critically reviewed the manuscript. EC performed some of the experiments. RM coordinated the study, performed experiments, analyzed the data and prepared the manuscript.
